# Local Hemostasis as the Critical Enabler for Safe Antithrombotic Therapy in Dentistry—Navigating Future Frontiers and Innovative Concepts

**DOI:** 10.3390/jcm15051823

**Published:** 2026-02-27

**Authors:** Diana Tatarciuc, Mioara Florentina Trandafirescu, Dragos Catalin Ghica, Iolanda Foia, Adina Oana Armencia, Irina Gradinaru, Magda Ecaterina Antohe, Lucian Stefan Burlea, Irina Mihaela Esanu, Roxana-Ionela Vasluianu

**Affiliations:** 1 Department of Medical Semiology, Faculty of Medicine, Grigore T. Popa University of Medicine and Pharmacy, 700115 Iasi, Romania; diana.tatarciuc@umfiasi.ro (D.T.); irina.esanu@umfiasi.ro (I.M.E.); 2 Department of Morphofunctional Sciences I, Grigore T. Popa University of Medicine and Pharmacy, 700115 Iasi, Romania; mio.trandafirescu@umfiasi.ro; 3 Department of Preventive Medicine and Interdisciplinarity, Faculty of Medicine, Grigore T. Popa University of Medicine and Pharmacy, 700115 Iasi, Romania; dragos.ghica@yahoo.ro (D.C.G.); iolanda.foia@umfiasi.ro (I.F.); 4 Department of Oral and Maxillofacial Surgery, Faculty of Dental Medicine, Grigore T. Popa University of Medicine and Pharmacy, 700115 Iasi, Romania; 5 Department of Prosthodontics, Faculty of Dental Medicine, Grigore T. Popa University of Medicine and Pharmacy, 700115 Iasi, Romania; irina.gradinaru@umfiasi.ro (I.G.); magda.antohe@umfiasi.ro (M.E.A.); roxana.vasluianu@umfiasi.ro (R.-I.V.); 6 Department of Management and Public Health, Faculty of Dental Medicine, Grigore T. Popa University of Medicine and Pharmacy, 700115 Iasi, Romania; lucianburlea@yahoo.com

**Keywords:** internal medicine, dentistry, concepts of administrations, anticoagulant, antiplatelet, protocols

## Abstract

Perioperative management of antithrombotic therapy in oral surgery represents an evolving paradigm. This critical review evaluates the contemporary scientific evidence that challenges the conventional practice of routinely discontinuing anticoagulants and/or antiplatelet agents to prevent postoperative bleeding. The traditional strategy carries an unacceptable risk of iatrogenic, sometimes severe, thromboembolic events. The aim of this systematic narrative review is to synthesize the current evidence (2015–2025) and to outline a new, patient-centered clinical framework that dynamically balances thromboembolic and hemorrhagic risks. Materials and methods: A systematic search of major databases (PubMed/MEDLINE, Scopus, Web of Science) identified relevant studies, structured according to the PICO framework. The search strategy prioritized high-level evidence, including clinical guidelines, systematic reviews, meta-analyses, randomized controlled trials, and prospective cohort studies published between January 2015 and November 2025. Results: The results reinforce an emerging consensus: the basis of safe management is the rigorous application of advanced local hemostasis techniques (e.g., prioritizing resorbable materials, sutures, topical hemostatic agents, and antifibrinolytics) and the use of perioperative decision-making algorithms. These measures allow, in most routine dental surgical procedures, the safe continuation of antithrombotic therapy, thus minimizing the thromboembolic risk without significantly increasing the risk of clinically significant bleeding. In the future, research should focus on optimizing materials science (novel biomaterials and controlled-release systems) and on standardizing and validating protocols in specific populations (e.g., patients on combination therapy or at extreme cardiovascular risk). This review argues that the adoption of this evidence-based model, with local hemostasis as a critical pillar, is essential for modern, ethical, and safe dental practice.

## 1. Introduction—The Paradigm Shift from Drug Cessation to Local Control

Over the past century, dentistry has strengthened its scientific foundation, prioritizing prevention and advanced diagnostic methods. While technical advances have brought undeniable improvements in clinical practice, some experts note that the basic principles of the discipline have remained relatively unchanged, emphasizing the importance of continuous revision of theoretical paradigms, not just technological [1,2,3]. In this context, the management of anticoagulant (AGT) and antiplatelet therapy (APT) represents one of the most complex clinical challenges today, given the risks associated with both the cessation and maintenance of these treatments. This necessitates a careful balance between managing bleeding risks and maintaining cardiovascular protection [4,5,6].

The range of anticoagulant medication is currently undergoing a significant transformation. We stand at the precipice of a new epoch, where the long-held paradigm of universal therapy interruption for dental procedures is being dismantled, replaced by a sophisticated, patient-centric doctrine of strategic peri-procedural management. The imperative to prevent thromboembolic events during dental procedures requires a fundamental reevaluation of traditional perioperative management [7,8,9].

The historical standard of care involved interrupting antithrombotic drugs prior to dental procedures to minimize bleeding risk. Antiplatelet therapy, important for preventing thrombotic events in patients with cardiovascular diseases, poses significant implications for dental treatment outcomes, particularly concerning bleeding risks during and after procedures [10]. The primary concern is the increased risk of hemorrhage due to impaired platelet function, which complicates oral surgical interventions such as extractions [11,12,13]. However, discontinuing antiplatelet therapy before dental procedures is generally not recommended, as it may elevate the risk of thrombotic events without significantly reducing bleeding complications during extractions [14,15,16]. Analysis of clinical studies, recognized for their methodological rigor, has shown that it is not necessary to suspend antiplatelet therapy before dental extraction procedures [17,18]. The cessation of antiplatelet therapy has been documented in case reports and series to potentially result in deadly outcomes, including stent thrombosis and myocardial infarction, though the absolute risk in dental populations specifically has not been quantified in prospective studies. In instances of surgery with elevated bleeding risk or patients with comorbidities that promote medication accumulation, a meticulous evaluation and tailored therapeutic regimen are recommended. However, a combined assessment of the specific antiplatelet drug, the patient’s risk of thrombosis, and the bleeding risk associated with the surgery remains essential [19,20].

In 2018, Miller highlighted that the common practice of discontinuing blood thinners for dental procedures is frequently unwarranted and may pose risks, advocating instead for local bleeding management while preserving anticoagulant therapy [21]. Despite the near-universal agreement among national medical and dental organizations and other specialists that anticoagulant therapy should generally be uninterrupted during dental procedures, there are still reasons for the temporary cessation of such therapy. An examination of these arguments reveals that they are founded on a compilation of myths and half-truths rather than on rational scientific conclusions [22,23,24]. The risk of embolic morbidity in patients whose anticoagulation is interrupted for dental surgery exceeds the risk of substantial bleeding complications in patients whose anticoagulation is maintained, even in cases of extensive surgery. A greater number of individuals use new-generation anti-thrombotic drugs; dentists need to stay up to date on how these drugs affect dental work and the optimum ways to stop bleeding [25,26].

The research by Ozyilmaz et al. [27] revealed significant deficiencies in the perioperative treatment of antithrombotic medicines. A significant number misidentified warfarin as an antiplatelet medication, and a considerable fraction indicated they regularly ceased therapy for patients with coronary stents. Consultant recommendations typically advise pre-procedural discontinuation, although seldom utilized established monitoring metrics such as the International Normalized Ratio (INR) for patients on direct oral anticoagulants [27]. Contemporary cardiology, neurology, and hematology guidelines, however, increasingly warn against this practice due to the heightened risk of stroke, myocardial infarction, or thrombosis [28,29,30].

Significant transformations in the pharmaceutical company, including the targeted application of direct oral anticoagulants (DOACs) and the meticulous management of vitamin K antagonists, necessitate that the medical field elucidates this distinction in medication administration, informed by authentic clinical proof. The primary focus has transitioned from broad protocols to comprehending pharmacokinetic constraints and employing novel local hemostatic drugs. As a result, the total discontinuation of therapy is now avoidable for the majority of patients due to developments in minimally invasive surgical techniques [31,32,33].

DOAC (direct oral anticoagulants) and NOAC (Non-Vitamin K Antagonist Oral Anticoagulants) refer to the same therapeutic class of oral anticoagulants. Both terms are used in the medical literature to refer to direct antagonists of coagulation factors (such as factor Xa or thrombin), as opposed to vitamin K antagonists (such as warfarin) [34,35,36]. The name NOAC was initially preferred to emphasize this different mechanism of action, but the term DOAC has gained ground, being considered more accurately descriptive, as it highlights the direct action on specific enzymes [37,38,39].

Systematic reviews and clinical trials now confirm that this strategy does not increase the risk of severe postoperative bleeding while eliminating the thromboembolic risks associated with bridging therapy [40,41,42].

A critical examination of the literature reveals meaningful differences in emphasis and specificity between guidelines issued by cardiology/hematology societies and those from dental organizations. Understanding these differences is essential for evidence-based practice and interdisciplinary communication.

This narrative review synthesizes the latest confirmations to provide a clear, evidence-based framework for safe and effective surgical therapy in this growing patient population. The evidence will be reviewed to establish that these local measures are not merely adjuncts but are the cornerstone of modern medical practice, providing a vital framework for improving clinical decision-making, particularly those involving surgical procedures, encouraging interdisciplinary collaboration, and ultimately optimizing patient safety and outcomes.

## 2. Materials and Methods

A search strategy was designed and executed to identify all relevant evidence pertaining to the management of patients on anticoagulant and/or antiplatelet therapy undergoing dental and oral surgical procedures. Studies were included according to pre-established criteria and structured according to the PICO framework.

This methodology was selected for three main reasons.

1. The objective is to synthesize a rapidly evolving field over the past decade (2015–2025) to identify paradigm shifts, conceptual frameworks, and future directions.
2. The evidence base includes heterogeneous study designs (guidelines, randomized clinical trials, observational studies) that do not lend themselves to quantitative meta-analysis.
3. The goal is to propose a new conceptual framework (risk-adapted stratified hemostasis) that synthesizes the existing evidence into a clinically applicable model.

This review does not perform a quantitative meta-analysis or formal assessment of risk of bias across all included studies, which defines it as narrative. This approach aligns with established definitions of narrative reviews that use systematic search strategies.

### 2.1. Search Strategy

The protocol was structured to align with the principles of rigorous evidence synthesis for a comprehensive narrative review. Electronic database searches were conducted in PubMed/MEDLINE, Scopus, and Web of Science (Clarivate). The search period was limited from 1 January 2015 to 1 November 2025, to capture the modern era of antithrombotic management. The search utilized a combination of Medical Subject Headings (MeSH) terms and keywords related to four core conceptual domains: (1) antithrombotic agents (e.g., “anticoagulants,” “antiplatelet agents,” “warfarin,” “direct oral anticoagulant,” “aspirin,” “P2Y12 inhibitors”); (2) dental procedures (e.g., “dental surgery,” “tooth extraction,” “oral surgical procedures,” “dental implant”); (3) perioperative management (e.g., “perioperative care,” “bridging anticoagulation”); and (4) outcomes (e.g., “postoperative hemorrhage,” “bleeding,” “thromboembolism,” “complications”). The reference lists of all included high-impact systematic reviews, meta-analyses, and clinical guidelines were manually screened to identify any additional pertinent studies.

### 2.2. Eligibility Criteria

Studies were selected according to pre-defined inclusion and exclusion criteria, formulated using the following framework.

Inclusion Criteria:

- Human patients (adults ≥18 years) receiving any form of chronic anticoagulation (e.g., vitamin K antagonists, DOACs, heparins) and/or antiplatelet therapy (e.g., single or dual antiplatelet therapy) who underwent any form of dental or oral surgical procedure.
- The perioperative management strategy for the antithrombotic agent(s), encompassing continuation, interruption (with or without bridging therapy), or modification of dosing.
- Any comparator, including different management strategies, placebo, or no intervention.
- Primary outcomes of interest were the incidence of postoperative bleeding (major and minor, as defined by study authors) and thromboembolic events (e.g., stroke, systemic embolism, myocardial infarction, stent thrombosis). Secondary outcomes included other surgical complications, need for re-intervention, and health-economic indicators when available.
- High-level evidence, including clinical practice guidelines from major professional societies (e.g., American College of Cardiology, American Heart Association, European Society of Cardiology, International Society on Thrombosis and Hemostasis, national dental associations), systematic reviews and meta-analyses, randomized controlled trials (RCTs), and prospective cohort studies.

Exclusion Criteria:

- Studies involving only animal models or in vitro experiments, unless they provided unique mechanistic insights critical for interpreting clinical findings.
- Non-dental surgical procedures (e.g., major orthopedic or cardiothoracic surgery), unless they included a direct comparative dental cohort or provided foundational management principles.
- Editorials, letters to the editor, and commentaries that did not present original data or a systematic analysis.
- Publications not in the English language, or for which a reliable translation was unavailable.
- Duplicate publications from the same patient cohort; only the most comprehensive or recent report was included.

## 3. Assessing the Hemostatic Mechanism Under Pharmacological Influence

Currently, the attitude towards anticoagulant therapy in the context of surgical interventions has changed radically. The simple practice of discontinuing antithrombotic medication has been abandoned, adopting a proactive approach that adjusts the coagulation process in a controlled manner. This new perspective sees the patient as a complex system; the drug does not simply “thin” the blood but causes a specific molecular blockage in the coagulation process, which can be managed.

### 3.1. Perioperative Management and Contemporary Evidence on DOACs, Novel Antiplatelets, and Pharmacogenomic Guidance

Advances in perioperative anticoagulant management have led to more refined strategies. Current practice considers evidence to decide whether to continue or discontinue DOACs or warfarin, based on the patient’s risk. This approach also uses reversal agents and pharmacogenomic personalization to balance the risk of clots with that of bleeding [43,44,45].

**Figure 1:**
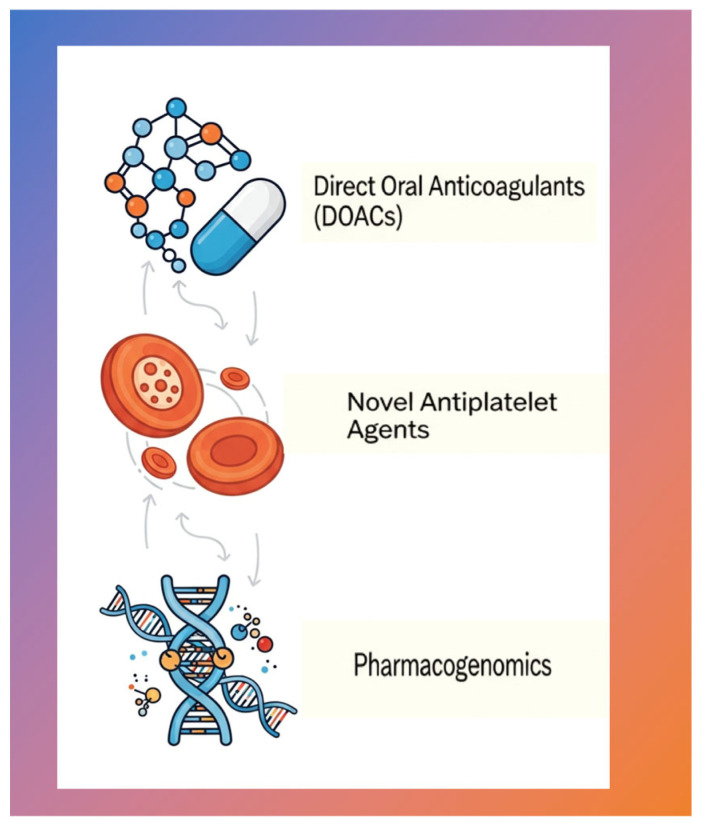
Contemporary pillars of perioperative antithrombotic management.

- DOACs—Emerging evidence supports a “timed interruption” strategy, taking advantage of their short half-lives. For procedures with low bleeding risk, omitting one dose (12–24 or ~30 h before the procedure) is usually sufficient, as demonstrated in studies such as the PAUSE trial, which showed low rates of major bleeding (1.8%) and thromboembolic events (0.4%) [46,47,48]. For the management of bleeding, specific bleeding reversal agents such as idarucizumab and andexanet alfa are now available, allowing for safer periprocedural management [49,50,51].
- Novel Antiplatelet Agents—Beyond P2Y12 inhibitors (e.g., clopidogrel), newer agents present challenges due to prolonged half-lives and irreversibility. Bridging is not feasible, underscoring the absolute reliance on local hemostasis [52,53].
- Pharmacogenomics (Current Reality and Future Potential)—Genetic testing (e.g., for CYP2C9 and VKORC1 variants) represents a scientifically advanced approach that can guide more precise, individualized warfarin dosing to achieve stable, low-therapeutic INR preoperatively, minimizing protocol deviations [54,55,56] (Figure 1).

### 3.2. Pharmacologic Disruption Points: Creating a Unique “Hemostatic Gap”

Each drug class creates a predictable, measurable deficit in this map, resulting in a characteristic hemostatic gap. The management of patients on antiplatelet therapy, particularly low-dose aspirin (LDA), who undergo dental extractions remains a common clinical concern, focused on balancing the thromboembolic risk with bleeding. Current evidence from systematic reviews and meta-analyses strongly supports the safety of continuing LDA monotherapy without interruption for routine dental extractions and minor oral surgical procedures in patients without additional bleeding risk factors [57,58,59]. This is corroborated by prospective studies demonstrating that LDA does not result in significant major post-extraction bleeding. Research indicates that bleeding is usually minimal and can be effectively managed with local interventions such as gauze pressure, hemostatic medications, or sutures [60].

The patient management becomes more complex with dual antiplatelet therapy (DAPT), usually aspirin combined with a drug such as clopidogrel. Although DAPT confers a higher risk of bleeding than monotherapy, studies suggest that the risk remains largely low. Research in patients with cardiovascular problems has found that although antiplatelet therapy increased the incidence of intraoperative and minor postoperative bleeding, the incidence of major bleeding requiring intervention in the study population was low [61,62,63]. It is essential to acknowledge that these data predominantly derive from single-tooth or limited extraction studies, and any generalization to more extensive procedures should be approached with caution. A meta-analysis specifically addressing DAPT concluded that dental extractions can be safely performed without changing therapy because the risk of significant bleeding is low and the risk of thromboembolism following discontinuation is substantial. Investigations into clot formation in DAPT patients confirm that although bleeding time may be prolonged, effective hemostasis is ultimately achieved [64].

Predicting patients who are likely to experience hemostatic difficulties is an area of investigation. A study using whole blood platelet aggregation assays suggested that this method could identify patients on antiplatelet therapy at higher risk of prolonged post-extraction bleeding, going beyond a common, generalized approach. Retrospective analyses align with prospective data, confirming that postoperative bleeding complications associated with antiplatelet drugs are rare and generally without serious consequences. Cross-sectional assessments further strengthen the paradigm that tooth extraction without modification of aspirin therapy is a standard and safe practice [65,66,67].

*Targeting Primary Hemostasis COX-1 Inhibitors (*e.g., *Aspirin)*: Aspirin irreversibly inhibits COX-1, blocking thromboxane A_2_ synthesis and reducing platelet aggregation for 7–10 days. This creates a fragile platelet plug susceptible to fibrinolysis [61,68,69]. DAPT adds P2Y_12_ receptor antagonism (e.g., clopidogrel), blocking ADP-mediated amplification and producing profound, synergistic platelet inhibition [70,71,72].

*Targeting Secondary Hemostasis, Vitamin K Antagonists (VKAs,* e.g., *Warfarin)*: Vitamin K antagonists act by inhibiting vitamin K recycling, a process essential for the activation of vitamin K-dependent coagulation factors (II, VII, IX, X) [73,74,75]. Blocking this cycle causes a gradual decrease in the level of active coagulation factors, reflected by an increase in the INR, functionally translating into reduced thrombin production and resulting in the formation of slow, weak, and structurally unstable fibrin clots [76,77,78];

*Targeted Thrombin or FXa Inhibition, Direct Oral Anticoagulants (DOACs)*

- Direct thrombin inhibitors (such as dabigatran) bind directly to the thrombin molecule, preventing its essential functions: the conversion of fibrinogen to fibrin, platelet activation, and the autoamplification of the coagulation cascade [79,80,81]. Thus, this class of drugs directly intervenes at the common final pathway, affecting both fibrin clot formation and platelet receptor (PAR) signaling [82,83]. Direct factor Xa inhibitors (such as apixaban, rivaroxaban, and edoxaban) bind to and directly block factor Xa activity, preventing the conversion of prothrombin to thrombin and thus prophylactically reducing the thrombin burst central to clot formation [84,85,86]. As a result, the amount of thrombin available to form and stabilize a clot is limited [87,88,89,90].
- Heparins UFH and LMWH exert their anticoagulant effect by potentiating antithrombin (AT), which inactivates the key enzymes of the coagulation cascade, thrombin (FIIa) and activated factor X (FXa). The mechanism differs between classes: UFH, through its pentasaccharide sequence, inhibits both enzymes equally, while LMWH has a predominant anti-FXa activity. As a result, the therapeutic effect is immediate after administration. This strong pharmacodynamic profile requires mandatory monitoring: the use of aPTT for UFH and anti-FXa levels for LMWH to optimize dosing and minimize the bleeding risk [91,92,93].

## 4. The Risk-Adapted Layered Hemostasis Framework

**Figure 2:**
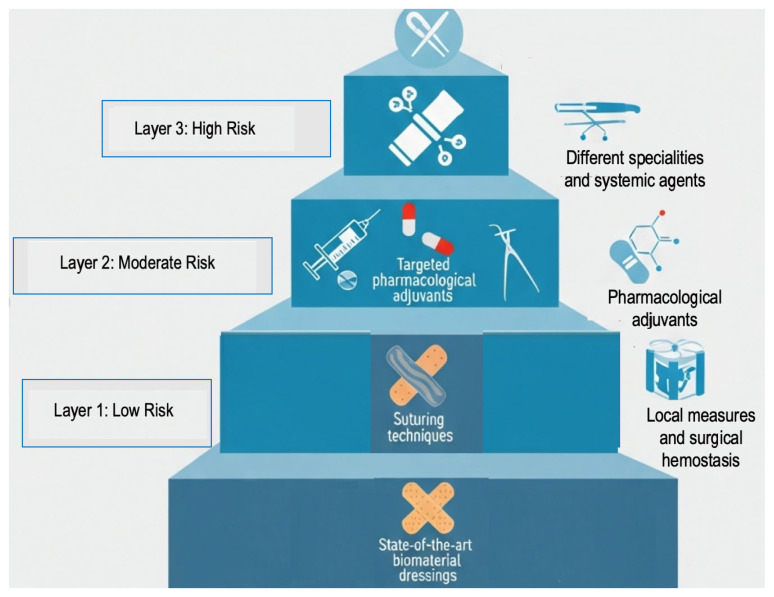
Scaling risk in wound management.

The Risk-Adapted Stratified Hemostasis framework is a new, evidence-based way to manage bleeding during and after dental surgery, especially for people taking antithrombotic medications. It moves away from the old “stop or continue” model and toward a more flexible, risk-stratified one. The main idea is that hemostatic interventions should be stratified in intensity, with each stratum responding directly to a specific patient type and procedural risk. This framework combines the latest information on drugs, biomaterials, and surgical interventions to make procedures safer and more likely to work (Figure 2).

Each layer preceding the last represents a higher level of overall risk, combining pharmacological, surgical, and biomaterial sciences [42,94].

It is proposed as a structured approach to clinical decision-making that integrates pharmacological risk assessment, procedural risk stratification, and evidence-based hemostatic interventions. Clinicians should apply this framework judiciously, recognizing that individual patient factors may necessitate deviation from the proposed schema. Prospective validation studies are needed to establish the framework’s safety, efficacy, and generalizability across diverse clinical settings.

### 4.1. Stratification of the Pharmacologic Hemorrhagic Risk

This stratification is based on the drug’s mechanism, pharmacokinetics, and the robustness of clinical evidence regarding bleeding risk in dental surgery.

#### 4.1.1. Low Pharmacologic Risk

Assessing perioperative bleeding risk is fundamental for safe dental practice in patients on antithrombotic therapy. A clearly defined low-risk category includes drugs with negligible influence on intraoral surgical hemostasis. For routine procedures, this primarily comprises patients on single-agent antiplatelet therapy (SAPT) with aspirin or a P2Y_12_ inhibitor (e.g., clopidogrel) alone. However, most evidence for P2Y_12_ inhibitor monotherapy is extrapolated from aspirin studies, as fewer studies specifically evaluate clopidogrel in dental surgery [95,96,97].

For patients on warfarin, an INR ≤3.5 measured within 24 h of the procedure is considered acceptable for continuation during routine dental extractions, as established by systematic reviews and multiple national guidelines [58]

Emerging agents such as cilostazol, a phosphodiesterase 3 inhibitor, show a postoperative bleeding risk profile similar to low-dose aspirin in early studies, classifying it as low risk pending further validation [98,99,100]. Established consensus guidelines recommend continuing these monotherapies, prioritizing enhanced local surgical hemostasis over modification of critical cardioprotective or cerebroprotective protocols, thereby balancing procedural safety with avoidance of life-threatening thromboembolic events [98,99,100].

#### 4.1.2. Moderate Pharmacologic Risk

Agents with significant hemostatic impact that require deliberate local interventions constitute this moderate-risk category [101,102,103].

Current evidence classifies patients treated with dual antiplatelet therapy (DAPT) or direct oral anticoagulants (DOACs) as a moderate risk, not a high risk, a distinction essential for evidence-based decision-making that favors personalized perioperative management over generalized therapy interruption.

A systematic review by Ockerman et al. (2020) [42] determined that although DAPT increases the risk of postoperative bleeding compared with monotherapy, bleeding is usually minor and manageable with local interventions [42]. One consideration that should be taken into account in routine practice is that the thrombotic risk from indiscriminate interruption outweighs the risk of bleeding [45,97]. Bensi et al. [101] concluded in their 2018 systematic review that although DOACs have a higher odds ratio for bleeding compared with well-managed warfarin, the absolute increase in risk in the included study is minimal [101]. This conclusion is based on observational data with heterogeneity in bleeding definitions and surgical complexity and should be interpreted with appropriate caution [104]. Yagyuu et al. (2017) [74] confirmed no significant difference in major post-extraction bleeding between patients treated with DOACs, warfarin, or no anticoagulant therapy when appropriate local protocols were implemented [74]. Therefore, the consensus supports the continuation of antithrombotic therapy in most cases, emphasizing that safe management relies on precise surgical technique and local hemostatic agents, rather than systemic pharmacological adjustments, in an interdisciplinary setting [105]. Classification into different therapeutic doses contributes to establishing a strict clinical treatment, influencing bridging strategies [54,106].

This classification recognizes its durable antithrombotic state and increased vulnerability to bleeding during invasive procedures [14]. Hamzah et al. [89], reviewing oral surgery in patients with ventricular assist devices, further emphasize the rigorous hemostatic protocols required for therapeutic anticoagulation, including LMWH, where perioperative management balances interruption of therapy to reduce bleeding while minimizing prothrombotic interruption [89].

#### 4.1.3. High Pharmacologic Risk

Current evidence indicates that patients on chronic vitamin K antagonist (VKA) therapy with stable therapeutic INR can safely undergo minor dental procedures without discontinuing anticoagulation, provided that rigorous local hemostasis is ensured using hemostatic agents, fibrin sutures, and antifibrinolytics such as tranexamic acid [58,107].

In contrast, a significantly elevated INR above the upper limit of the therapeutic range (specifically, values > 4.0) is a clinical marker of a pronounced hypocoagulable state and is recognized as a major risk factor for intra- and postoperative bleeding events, based on the consensus guidelines of the British Committee for Standards in Hematology and the American College of Chest Physicians [108,109].

Management of intrinsic coagulopathies, such as hemophilia, requires increased vigilance. These patients not only have pharmacologically altered hemostasis but also have a fundamental deficiency in the proteins of the coagulation cascade. The evidence guiding dental management in this population comes primarily from small case series and extrapolation from the hematology literature, representing low-certainty evidence. Dental procedures performed in this population, particularly those requiring therapy with bypass agents (e.g., recombinant factor VIIa or activated prothrombin complex concentrates) to temporarily correct the coagulopathy, carry a significant risk of severe, delayed bleeding. Based on the available evidence and expert consensus, these therapies are not appropriate for the outpatient dental setting and require inpatient treatment with hematological monitoring [110].

Management of high-risk pharmacological bleeding is based on three pillars: risk stratification (differentiating between triple therapy, supratherapeutic INR, and congenital conditions), multidisciplinary collaboration to determine antithrombotic regimens, and procedural rigor that integrates targeted systemic correction with strict local hemostatic techniques [111].

### 4.2. Stratification of the Procedural Hemorrhagic Risk

#### 4.2.1. Low-Trauma Procedures

Advances in antithrombotic therapy require an evidence-based approach to assessing the risk of bleeding in minimally invasive dental procedures. Recent studies challenge the traditional belief of a constant and increased risk of bleeding, advocating a more nuanced assessment that distinguishes between the complexity of the procedures and the pharmacological agents used [112,113].

In 2019, Berton et al. [106] presented critical data in this review, directly contrasting DOACs with vitamin K antagonists (VKAs) in the context of uncomplicated single-tooth extraction, a fundamental minimally invasive surgical procedure [106]. Analysis of the data obtained determined that the risk of postoperative bleeding for patients receiving DOACs was not significantly different from the results obtained for those receiving VKAs [109]. This statement fundamentally challenges the clinician’s tendency to consider newer anticoagulants as more dangerous, especially in less complex procedures. The study highlights that, in the case of uncomplicated dental extractions, specific oral anticoagulants may be less significant than previously anticipated when appropriate local hemostatic techniques are diligently implemented.

In 2021, AlSheef et al. [111] further confirmed the notion of procedural stratification by investigating postoperative hemorrhage after dental extractions in patients undergoing various antithrombotic therapies [111]. Their data confirm that the occurrence of clinically significant bleeding remains minimal, especially in cases with minor surgical trauma. The research supports a risk matrix that emphasizes the invasive nature of the proposed surgical intervention, such as single-tooth extraction compared to complex osteotomies, alongside pharmacological considerations. Minimally invasive techniques reduce the initial bleeding risk, facilitating effective perioperative management without the need for interruption of therapy [114].

Contemporary methodology in this field creates a stratification of the risk of complications according to the severity of the procedure. The literature supports the continuation of antithrombotic medication during minimally invasive surgical interventions, accompanied by correct local hemostasis techniques, absorbable sponges and sutures, or the use of tranexamic acid mouthwash. This paradigm shift improves patient safety by mitigating the thromboembolic risks associated with drug discontinuation and aligns surgical practice with demonstrated clinical evidence, promoting a standardized, safe, and patient-centered approach to common dental procedures [40,115].

#### 4.2.2. Procedures Involving Many Injuries or Extensive Vascular Networks

Clinical studies confirm that major surgical procedures clearly carry a high risk, while flap surgeries, recommended in periodontal surgery, require more careful delineation. Synthesized evidence from recent cohort studies and meta-analyses indicates that, for these procedures, procedural risk interacts with patient-specific pharmacological and hematological factors.

The research by Ohba et al. [116] established that, in minor oral surgery, procedural complexity, quantified by factors such as the number of extractions, increases the baseline risk [117]. This is critically contextualized by subsequent research demonstrating that intraoperative bleeding serves as a significant predictor of postoperative hemorrhage, as noted by Rocha et al. [118]. Therefore, the classification of a procedure as “moderate” depends on its potential to cause sustained intraoperative vascular injury, which may challenge hemostatic mechanisms. This procedural risk is then potentiated by specific pharmacological regimens. Iwata et al. found that direct-acting oral anticoagulants (DOACs), especially at higher doses, and warfarin with an INR > 3.0, confer a significantly increased risk compared with well-controlled warfarin [119]. Concurrently, a meta-analysis by Villanueva et al. confirms that dual antiplatelet therapy possesses a significantly higher risk than single-agent therapy [117].

The highest-risk cohort includes patients treated with high-dose DOACs, supratherapeutic warfarin, or dual antiplatelet therapy undergoing multi-dental or bone surgery. A moderate-to-high-risk level includes patients receiving standard-dose DOACs or single antiplatelet agents (e.g., clopidogrel) for similar procedures. Finally, patients receiving well-controlled warfarin monotherapy (INR in the therapeutic range) or aspirin represent a moderate-risk group in which procedural factors become paramount. This stratification argues against universal discontinuation of therapy, instead advocating for perioperative strategies based on risk levels, from meticulous local hemostasis for moderate-risk patients to careful pharmacological management for high-risk individuals, always within the framework of interdisciplinary consultation.

#### 4.2.3. High-Trauma Procedures

In 2020, the pivotal study by Bajkin et al. [120] defined this implant surgery as a procedure with a high risk of bleeding due to its invasive characteristics, large wound surface area, and frequent inability to achieve primary closure or adequate local hemostatic pressure [120].

In 2024, Kang et al. presented significant data indicating that even a minimally invasive technique, such as immediate flapless implant placement, does not significantly reduce the risk of bleeding in antithrombotic patients [121]. Miziara et al. [122] demonstrated that although continuous antithrombotic therapy is usually safe for uncomplicated extractions, increased caution is recommended in the planning of the intervention in the case of implant surgery. The specific approach and surgical trauma further increase this risk stratification [122]. This emphasizes that the potential for bleeding is caused by biological trauma rather than flap design itself.

Furthermore, thorough risk stratification must also include systemic pathophysiology. Patients with severe renal disease, as demonstrated by Mochizuki et al. [123], present a multifaceted hemostatic challenge resulting from platelet dysfunction and compromised vascular integrity. This evidence, derived from a single-center retrospective analysis, suggests that even standard oral surgical procedures may result in significant bleeding in this population. While prospective validation is limited, current expert consensus indicates that major traumatic surgeries should ideally involve thorough preoperative optimization and interdisciplinary management when clinically feasible [123]. A comprehensive classification model incorporates two fundamental dimensions: procedural trauma, such as implant surgery and complex surgical procedures, representing the high-risk category, and the patient’s inherent coagulopathic predisposition resulting from antithrombotic medication or systemic conditions [124,125,126]. The literature emphasizes the need for accurate classification of surgical procedures, such as dental implant surgery, as having a high risk of bleeding. Effective care is based on this classification, guiding physicians towards personalized therapies, such as time optimization, aggressive local hemostatic measures, and mandatory interdisciplinary consultation, thus balancing thromboembolic prophylaxis with the consequences of surgical bleeding [127,128,129].

### 4.3. Application of the Layered Hemostatic Plan

The RALH matrix recommends combining agent categories from three distinct layers, each addressing a specific need.

#### 4.3.1. Foundation Layer: Applied to All Procedures

In line with the hemostasis management strategy established in 2025 by Iijima et al. [127] for chronic gingival bleeding, the RALH matrix provides a systematic, multifaceted methodology for achieving hemostasis in complex surgical and traumatic lesions. This paradigm transcends the rudimentary use of a single agent, advocating a systematic fusion of hemostatic categories into three separate, complementary phases, each addressing a specific pathophysiological requirement at the surgical wound level [127].

The hemostatic layer, also the first layer, is composed of substances such as collagen, gelatin or cellulose-based products, whose main function is to provide a physical tamponade effect. Their role is to aggregate platelets and stimulate coagulation factors. The success of successive layers depends on the initial stabilization of the wound microenvironment, which reduces blood flow [130].

The adhesion layer required to fix hemostatic structures and improve blood clot stabilization is formed by fibrin sealants or synthetic tissue adhesives, such as cyanoacrylates. Their function is to ensure good adhesion to the wound edges and to be involved in the later stages of the coagulation mechanism. This layer creates a flexible and impermeable membrane that reduces the risk of late hemorrhage caused by mechanical stress or fibrinolysis, providing protection against external pollutants [131,132].

The last layer formed is intended for situations of persistent or recurrent bleeding. This layer involves the use of potent systemic or topical procoagulants, including high-concentration thrombin or antifibrinolytics.

#### 4.3.2. Augmentation Layer: Pharmacologic Risk-Guided

- Contemporary management of patients on chronic antithrombotic therapy undergoing invasive dental procedures requires a paradigm shift from generic compression to a mechanism-based, targeted hemostatic approach. This strategy, termed the Stratified Hemostatic Plan, advocates the selection of topical adjuvants based on an assessment of pharmacological risk that directly counteracts the specific adverse effect of the anticoagulant or antiplatelet [51].
- For patients receiving direct oral anticoagulants (DOACs), classified as having moderate pharmacological risk, the therapeutic goal is to bypass the pharmacologically inhibited factor. DOACs directly inhibit Factor Xa or thrombin (Factor IIa), halting the propagation phase of the coagulation cascade. Consequently, agents that rely on an intact intrinsic or extrinsic pathway are suboptimal. The ideal strategy utilizes a physiological agent, such as a thrombin-gelatin matrix. Accordingly, the strategic goal is to enhance and accelerate the development of the first platelet plug. This is best achieved with a contact activator, such as a collagen sponge or microfibrillar collagen. Randomized clinical trials indicate that these materials create a potent thrombogenic surface that directly promotes adherence, activation, and release of granular contents, thereby effectively enhancing the primary hemostatic phase despite diminished platelet activity. Recent prospective studies further validate the efficacy of other hemostatic dressings, such as chitosan, which similarly promote platelet aggregation and adherence to the injury site [133].
- The most complex scenario involves patients treated with vitamin K antagonists (VKA), who often present a moderate to high pharmacological risk due to functional deficiency of multiple coagulation factors (II, VII, IX, X). While traditional agents such as oxidized cellulose, which form an artificial seal, are commonly used, some investigators suggest that the most physiologically consistent strategy within the layered plan may be the use of a physiological agent (thrombin-gelatin). However, direct comparative evidence supporting this preference over traditional agents in VKA patients is limited, and clinical decisions should consider availability, cost, and practitioner experience. Systematic studies of localized hemostatic measures validate the essential function of these personalized therapies in achieving safe hemostasis in this demographic. Systematic, risk-based methodology, based on physiological principles and growing clinical evidence, aims to enhance the safety and efficacy of dental surgical hemostasis in patients with complicated medical conditions [134,135].

#### 4.3.3. Stabilization Layer: Procedure-Guided Approach Based on Anatomical and Functional Requirements

- The integrity of the initial fibrin clot, necessary for the healing of postsurgical wounds, is strictly dependent on the enzymatically active oral cavity environment, which also contains a vast microbial flora. Therefore, contemporary surgical protocols emphasize the strategic application of specific sealing and stabilizing agents to create an optimal environment for regeneration. This approach is fundamentally guided by a detailed analysis of anatomical and functional requirements, moving beyond a universal methodology towards a personalized therapeutic intervention.
- In the posterior regions, especially at the molar level, functional challenges are pronounced due to the complex anatomy, combined with significant masticatory forces, tongue movement, and the constant presence of oral fluids that create a high-risk environment for the development of alveolitis or for the destruction of the blood clot. In such situations, the main objective is to establish a durable physical barrier [53].
- In situations with generalized suppuration or in patients with underlying coagulopathy characterized by hyperfibrinolysis, a condition in which the fibrin matrix of the clot is prematurely degraded, a simple local barrier is ineffective. The central pathological process is rapid enzymatic dissolution of the clot, often exacerbated by inflammatory factors. For these complex cases, procedural guidelines are directed towards achieving sustained local hemostatic and antifibrinolytic activity. This is best achieved by the use of an impregnated support matrix.
- Filling the postoperative wound with an absorbable gelatin sponge or surgical gauze saturated with tranexamic acid (TXA) is an appropriate therapeutic intervention in such situations. TXA, a synthetic lysine analog, competitively inhibits the activation of plasminogen to plasmin, the key enzyme in fibrinolysis. By delivering the agent directly to the alveolus via a carrier, a high local concentration is maintained, providing prolonged antifibrinolytic activity. This method ensures stabilization of the fibrin network against premature degradation. Topical administration limits the pharmacological effect, resulting in negligible systemic absorption and an exemplary safety profile, a consideration of major importance for patients on complex anticoagulant regimens, as highlighted in contemporary reviews of periprocedural bleeding management.
- The algorithmic selection between a passive and active sealant, drug-impregnated stabilizer, is influenced by a clear assessment of anatomical structures versus biological risks. This site-specific, procedure-guided paradigm ensures that therapeutic interventions are tailored to clinical requirements, thereby protecting the clot, increasing patient comfort, and promoting predictable bone healing [42,136,137] (summarized in Table 1).

Periprocedural management of antithrombotic drugs is based on key pharmacological principles that determine bleeding risk and drug clearance. This table provides a consolidated reference for half-lives, elimination pathways, and recommended timing of the last dose before surgery for the most commonly used agents (summarized in Table 2).

## 5. The Hierarchy and Technical Detail of Critical Local Measures

A systematic, hierarchical strategy in which each procedural layer serves a function is paramount, from fundamental planning to extended postoperative care. This approach is increasingly informed by pharmacogenomics, biomaterials science, and a refined understanding of the oral cavity environment. Chronic oral inflammation driven by dysbiotic biofilm impairs vascular integrity and healing. The role of chlorhexidine is being reevaluated beyond simply reducing bacterial flora, which has the role of reducing inflammatory mediators (e.g., PGE2, IL-1β) at the surgical site, thereby stabilizing the microvascularization and reducing the predisposition to secondary bleeding.

### 5.1. Procedural Risk Stratification: A Clinical Classification System

To complement the assessment of pharmacological risk, surgical procedures are classified according to their bleeding risk. Clinicians can tailor hemostatic interventions to the intensity of procedural trauma.

Dental medical procedures that present a low risk of bleeding include:

- Supragingival prophylaxis and scaling;
- Administration of local anesthesia (including nerve blocks);
- Minor soft tissue procedures (frenectomy, small excisional biopsies <0.5 cm);
- Placement of orthodontic bands/brackets;
- Endodontic treatment (access cavity preparation, root canal instrumentation) limited to the pulp chamber;
- Single, uncomplicated tooth extraction (mobile teeth, simple forceps extraction);

These procedures involve minor vascular changes, a limited wound area, and hemostasis is usually achieved by gauze compression, the standard technique used postextractionally. The risk of clinically significant bleeding requiring intervention approaches that of non-antithrombotic patients.

Procedures with moderate risk of bleeding include:

- Alveoloplasty (limited, involving 1–2 alveolar sites);
- Apicoectomy (anterior teeth);
- Soft tissue biopsies requiring suture closure;
- Multiple dental extractions (2–3 teeth) in the same quadrant;
- Surgical extraction of impacted teeth requiring osteotomy;
- Flap periodontal surgery (open debridement, crown lengthening);
- Placement of a single dental implant with flapless or minimal flap techniques.

These procedures create significant vascular lesions, especially in flap interventions, alveolotomy, or procedures involving manipulation of well-vascularized tissues. Complications may occur in clinical situations where effective primary closure is not achieved. These cases require the planned application of advanced hemostatic agents beyond basic measures.

Procedures with a high risk of bleeding are:

- Periodontal surgery involving bone resection;
- Multiple tooth extractions (>3) with extensive alveoloplasty;
- Surgical resection of intraoral pathology (torus removal, large cystic lesions);
- Procedures in highly vascularized anatomical regions (posterior palate, floor of mouth, retromolar area);
- Full-arch or multi-quadrant implantology (≥4 implants);
- Complex bone graft procedures (en bloc grafts, sinus lift with lateral window);
- Any surgical procedure requiring general anesthesia with a high risk of immediate postoperative bleeding.

These cases require interdisciplinary consultation and consideration of perioperative timing optimization. In patients with moderate to high pharmacological risk, such procedures may warrant postponement for medical optimization or hospitalization (summarized in Table 3).

### 5.2. Surgical Technique

Effective management of intra- and postoperative bleeding is a cornerstone of safe oral surgical practice, especially in an era when patients are increasingly treated with various anticoagulant and antiplatelet regimens without interruption of therapy. The paradigm for preventing bleeding complications does not begin with adjuvant agents but with meticulous, minimally traumatic surgical technique, establishing what is called primary hemostasis. This fundamental approach is essential, as evidenced by studies demonstrating that dental extractions can be safely performed in patients taking medications such as direct oral anticoagulants (DOACs), vitamin K antagonists (VKAs), and antiplatelet drugs when supported by rigorous local measures [8,138].

#### 5.2.1. Primary Surgical Control: The Minimally Traumatic Philosophy

Vascular changes that occur during surgery can be largely caused by the surgical technique used. A recent approach to extraction techniques, the use of piezosurgery or microrotation systems, has made a major contribution to preserving bone tissue by preserving its integrity. The atraumatic technique also represents a strategy for primary hemostasis due to the minimization of tissue damage and vascular disruption. This meticulous approach directly addresses the surgical lesions, reducing the burden of initial hemorrhage that subsequent local measures must manage, a concept supported by clinical studies [102,139].

#### 5.2.2. Intra-Socket and Wound Management: Ensuring an Optimal Hemostatic Environment

After tooth extraction, direct management of the surgical wound is the next critical phase for primary control. Systematic curettage is essential for the removal of granulomatous tissue and pathological epithelial debris. This maneuver ensures that wound closure and healing occur from a base of healthy vascular tissue with optimal regenerative capacity.

Primary wound closure by suture, when anatomically feasible, serves multiple hemostatic functions. It stabilizes the mucoperiosteal flaps, approximates the wound edges to reduce the physical volume of the clot required for stabilization, and creates a physical barrier that prevents food impaction and subsequent clot disruption. The efficacy of such precise surgical management is evidenced in randomized clinical trials evaluating hemostasis in patients on antiplatelet therapy, where controlled surgical technique forms the basis against which additional local hemostatic agents are evaluated [42,101].

#### 5.2.3. Immediate Post-Extraction Tamponade: Socket Compression

The final stage of the intraoperative protocol is immediate postoperative care. Immediate application of pressure remains the standard, non-pharmacological procedure. This is achieved by compressing the alveolus with a sterile gauze pad held under firm, continuous pressure by the patient for 20–30 min to 2 h. This simple but effective technique provides initial hemostasis by facilitating platelet aggregation and activation of coagulation mechanisms [61,68].

Surgical management of bleeding is a sequential protocol initiated by the skill of the operator. A philosophy of minimally invasive surgery, using precise instruments and techniques, directly reduces vascular damage. This is strategically followed by preparation of the alveolus and primary wound closure to optimize the local biological environment for the initiation of the coagulation process. The surgical technique addressed is directly involved in the management of patients, especially those taking antithrombotic medications, preceding the application of topical hemostatic biomaterials as secondary adjuvants [140].

### 5.3. Advanced Local Hemostatic Agents

#### 5.3.1. Absorbable Hemostatic Sponges/Gelatins

Postoperative bleeding can be managed by the application of hemostatic adjuvants. Among these, absorbable gelatin-based sponges, such as Gelfoam^®^ and Surgifoam^®^, occupy a critical position, acting as bioactive structures.

Their main mechanism of action is blood saturation; the porous, hydrophilic matrix of these materials provides a large, three-dimensional surface area that actively sequesters platelets and concentrates coagulation factors, thereby accelerating the intrinsic pathway of clot formation [101,136]. This process effectively creates a localized, hardened hemostatic plug directly at the site of vascular injury, a principle confirmed by clinical evaluations comparing their efficacy with other topical agents [141]. The therapeutic implementation of these materials is supported by a set of studies evaluating their efficacy in high-risk situations [127].

#### 5.3.2. Topical Thrombin

Its mode of action is the direct conversion of fibrinogen to fibrin to form a stable, native clot mesh that remains functional despite systemic anticoagulation. Topical thrombin is an important local hemostatic agent in dental surgery, especially for patients taking direct thrombin inhibitors (DTIs). It bypasses the pharmacological blocker implemented by DTIs, which prevents the action of soluble thrombin [142,143,144].

#### 5.3.3. Fibrin Sealants (Two-Component)

Fibrin sealants are composed of two components, specifically designed to replicate the physiological endpoint of hemostasis, functioning independently of the patient’s coagulation pathway, bypassing potential deficiencies in coagulation factors. While multiple case series and small randomized trials support their efficacy, it is important to note that large-scale, multicenter RCTs specifically in dental surgery patients on antithrombotic therapy are lacking. The evidence supporting fibrin sealants in this context is therefore graded as moderate, based on consistent findings from smaller studies and extrapolation from the general surgical literature [145,146,147].

#### 5.3.4. Tranexamic Acid

Topical application of tranexamic acid (TXA) represents a targeted pharmacological strategy for the control of post-extraction hemorrhage by local action in the distinct fibrinolytic environment of the oral cavity [148,149,150]. The mechanism of action is based on the local inhibition of plasminogen activation, thus neutralizing the effect of salivary activators that induce premature degradation of the fibrin clot. In clinical practice, it is frequently used as a solution for oral irrigations (4.8–5%), administered in the pre- and/or postoperative period, or by local application with impregnated gauze in the post-extraction site [113,151,152,153]. This method of administration ensures high therapeutic concentrations at the site of hemorrhage, limiting systemic absorption. The survey by Chinnaswami highlighted that dentists often lack comprehensive knowledge about managing patients on oral antithrombotic medications, underscoring the need for reliable topical hemostatic options like this one [154]. 

Data obtained from randomized studies in the dental surgery population attest to a hemostatic efficacy comparable to or superior to other topical agents, especially in patients under anticoagulant or antiplatelet therapy, with available evidence suggesting no significantly increased thromboembolic risk when used topically, though studies are not sufficiently powered to detect rare thrombotic events [155,156,157].

#### 5.3.5. Oxidized Regenerated Cellulose (e.g., Surgicel^®^)

Oxidized regenerated cellulose (ORC), like Surgicel^®^, is a flexible topical hemostatic agent, very effective in dental surgery for patients with coagulation problems. In contact with blood, it provides a low-pH mechanical mesh that promotes clotting and can be left in situ to be absorbed. This acidic environment is bactericidal in nature, which confers a post-surgical advantage [141,158,159,160].

##### Platelet-Rich Fibrin (PRF)

**Figure 3:**
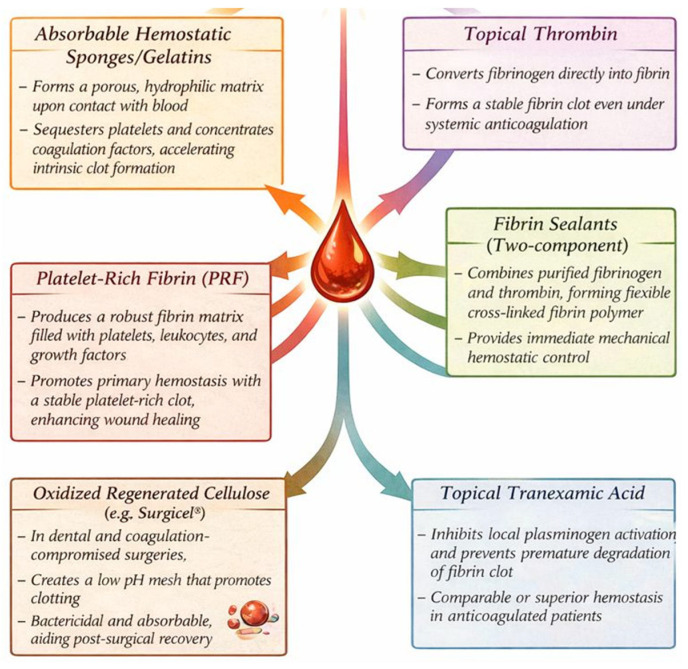
Local hemostatic agents.

Platelet-rich fibrin (PRF) is an autologous biomaterial that plays a key role in both stopping bleeding, especially in patients undergoing antithrombotic drug treatment. PRF works by producing a robust fibrin matrix containing platelets, leukocytes, and growth factors. This matrix actively promotes primary hemostasis by causing platelets to adhere to each other and generating a stable clot [161,162,163]. The continuous release of bioactive molecules accelerates bone healing, ensuring a favorable postsurgical prognosis. Clinical trial data indicate that the application of this autologous material in oral surgical procedures, such as tooth extraction and implant placement, could mitigate the risks of bleeding without requiring the discontinuation of anticoagulant or antiplatelet medication. Recent research indicates that these advantages extend to more complex treatments, such as sinus floor augmentation, reducing the incidence of early failures [164,165,166,167] (Figure 3).

A stratified description of the selection of hemostatic agents, distinguishing the “gold standard” from the “acceptable alternatives,” is a necessity in terms of clinical case management. For low-risk procedures, absorbable gelatin sponge with suture (the gold standard) can be replaced by gauze pads alone. In moderate-risk cases, the gold standard combination of absorbable matrix and tranexamic acid (TXA) rinse is extremely cost-effective, but oxidized cellulose with primary closure is also an acceptable alternative. For high-risk procedures, the best recommendation is appropriate patient scheduling, oxidized cellulose closure, topical administration of crushed TXA, and mandatory postoperative monitoring. A cost-effective autologous alternative is PRF, which requires the purchase of a special centrifugation system. For practices that perform regular surgeries with high bleeding risk, this investment may prove economically advantageous over time while providing the benefits of autologous growth factors [95,97,113].

## 6. Discussion—How Local Measures Make Drug Continuation Safe?

This review is structured into three thematic parts. Part 1 establishes the paradigm shift from drug discontinuation to local hemostatic control and provides the basis for understanding the hemostatic gaps specific to each drug. Part 2 systematically stratifies pharmacological and procedural bleeding risks, integrating them into the new Risk-Adapted Stratified Hemostasis (RALH) framework, a three-tiered interventional strategy. Evidence-based guidelines on surgical techniques and advanced hemostatic agents are provided in Part 3, concluding with a discussion of how local measures allow for the safe continuation of drug administration, an assessment of the quality of the evidence, and standardized postoperative protocols.

The oral cavity is a uniquely challenging environment for hemostasis, and the oral environment is uniquely hostile to clot formation. Saliva, while containing some pro-coagulant factors, is overall fibrinolytic and acts as a constant diluent. The wet, mobile, and microbially rich field prevents wound drying and crust formation, a key step in healing. Sutures alone cannot address these fluid-dynamic and biochemical challenges. Local agents are therefore designed to adhere to moist tissues, resist washout, and either neutralize fibrinolysis or provide an artificial extracellular matrix resistant to enzymatic degradation [168,169].

The most immediate function of local hemostatic agents is the provision of a site-specific physical barrier, facilitating natural clot formation within the protected niche, independent of systemic coagulation status. It creates a protected physical niche where the initial phases of the coagulation cascade can proceed, even in the presence of systemic anticoagulants that inhibit soluble clotting factors. They shield the platelet plug from the disruptive action of saliva and oral microflora, allowing it to mature into a stable fibrin clot. Materials such as gelatin sponges, oxidized cellulose, or collagen plugs conform to the surgical site, exerting direct mechanical pressure on severed capillaries and venules [170]. This tamponade effect reduces blood flow, facilitates the approximation of vessel walls, and provides a three-dimensional scaffold for clot formation and operates independently of the patient’s systemic coagulation status.

The healing of the post-extraction wound can be influenced by (1) the mechanical action of the tongue and musculature, (2) the proteolytic and fibrinolytic enzymes present in saliva, and (3) the colonizing oral microflora, which can both directly degrade the clot and incite inflammatory processes. Agents like fibrin sealants or thrombin-soaked gels initiate the final common pathway of the coagulation cascade at the wound surface itself. When combined with the patient’s own platelets and cofactors, they generate a stable fibrin clot in situ. Targeted administration achieves high local efficacy while avoiding systemic drug interactions or side effects [171,172,173].

In patients on anticoagulant therapy who undergo routine or minor dental procedures, the major clinical risk is hematoma formation. This potential complication requires careful and personalized surgical management that balances the efficacy of the procedure with patient safety, avoiding dangerous bleeding events [174,175].

Babaji et al. [18] confirmed that antiplatelet monotherapy or even dual antiplatelet medication does not require any modification or interruption before minor oral surgery. Most postoperative bleeding can be effectively managed with local hemostatic interventions [18]. In the study by Olmos-Carrasco et al. [176], 8.3% of patients undergoing dual antiplatelet therapy experienced bleeding complications more than 30 min after dental extractions, which were managed with local hemostatic techniques. The results of this study confirm that dual antiplatelet therapy can be continued during dental surgery [176]. Due to the different modes of action of antiplatelet and antithrombotic drugs, the risk of bleeding may be significantly higher in patients who discontinue such therapy, suggesting that continued medication may be safer. DOACs and VKAs have similar safety profiles without routine discontinuation for low-risk extractions [177].

Studies suggest that dual antiplatelet therapy increases the risk of postoperative bleeding compared with single-agent or no medication, while local hemostasis effectively managed all bleeding incidents, with no major clinically significant outcomes reported. Although anticoagulant therapy is important for preventing thromboembolic events, it presents challenges in dental practice. Some practitioners may advocate for more conservative approaches, emphasizing the need for individualized treatment plans that consider both anticoagulant and dental health needs. The proven efficacy of robust local protocols has allowed the development of universal guidelines recommending continued anticoagulant medication, simplifying decision-making and improving safety [178,179,180].

By providing a precise way to manage bleeding, these measures dissociate oral surgical risk from the patient’s systemic drug history. This concept underlies the recommendation by cardiology, hematology, and dental societies against discontinuing anticoagulant and antiplatelet therapy for minor or flap-related dental procedures. This paradigm minimizes the much higher risk of thromboembolic events associated with drug discontinuation, simplifies preoperative decision-making for the dentist, and standardizes postoperative care. Local hemostasis thus becomes the cornerstone of a protocol focused on patient safety, reducing morbidity, and improving outcomes across the entire patient population [181,182].

**Figure 4:**
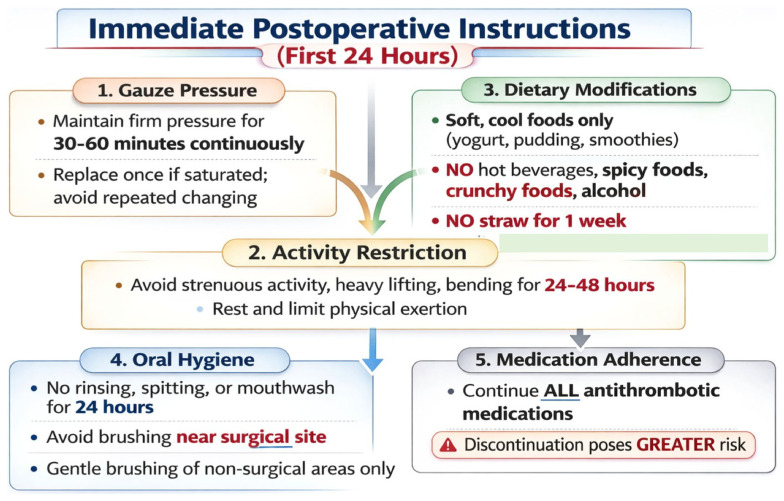
Standardized postoperative protocol.

The success of intraoperative hemostatic measures critically depends on meticulous postoperative care and patient adherence to specific instructions. The oral environment, characterized by constant moisture, biomechanical forces developed during speech and mastication, and fibrinolytic activity, requires a structured approach to postoperative management that extends beyond generic post-extraction advice (Figure 4).

Despite the evolution of evidence-based guidelines over the past decade, significant gaps persist among dental practitioners regarding perioperative antithrombotic management. Currently, point-of-care genetic testing remains largely inaccessible to general dentists due to several significant barriers:

1. Cost and reimbursement—Genetic testing panels typically cost $200–500, with inconsistent insurance coverage and no specific reimbursement codes for dental procedures.
2. Processing time—Results require 3–7 days from reference laboratories, excluding decisions on same-day procedures or medical emergencies.
3. Interpretation complexity—Genotype-guided dosing requires specialized pharmacokinetic knowledge and access to clinical decision support tools that are not typically available in dental offices.

### Pathway to Clinical Adoption

The integration of pharmacogenomics into routine dental practice will likely follow a phased approach:

- Primarily used in university medical centers and hospital dental offices, where integrated electronic medical records and pharmacy services exist. Genetic information, when already available in patient records, can inform perioperative planning.
- Development of direct-to-provider test panels with rapid turnaround times (24–48 h) and simplified interpretive reports. Integration into preoperative assessment protocols for patients requiring high-risk surgery (complex procedures, unstable INR).
- Widespread availability of low-cost genetic screening as part of comprehensive health assessments.

Dentists should now recognize pharmacogenomics as an emerging tool that, when available, will optimize anticoagulation management. However, at present, its absence does not preclude treatment of the patient in the dental office. The fundamental foundations of current management remain risk stratification, procedural planning, and systematic application of local hemostatic measures.

## 7. Educational and Collaborative Objectives

### 7.1. Role of Dental Education in Preparing Students to Manage Patients on Antiplatelet/Anticoagulant Therapy

The traditional focus on simple drug name recognition is insufficient. As has been observed in research conducted to date on the knowledge and attitudes of dental students, there is often a significant discrepancy between theoretical knowledge and clinical application [25]. A survey by Alabdulkarim et al. [183] of dental students and trainees found that while most recognized warfarin (92.9%) and aspirin (86.1%), awareness of the newer direct oral anticoagulants, such as rivaroxaban (10.7%) and apixaban (8.2%), was strikingly low. Critically, only half (49.5%) correctly identified the safe INR threshold (<4.0) for extractions, and only 37% knew the minimum safe platelet count (50,000/μL). Furthermore, over half (52%) of participants believed that antiplatelet or anticoagulant medications should be stopped even for minor surgical procedures such as single-tooth extraction, a practice directly contradicted by current evidence due to the associated thromboembolic risk [183]. Dental education must go beyond drug recognition, teaching students not only that they should use topical measures but also why specific agents are selected based on a drug’s mechanism of action.

### 7.2. Importance of Continuing Professional Development for Practicing Clinicians

For practicing physicians, the rapid evolution of antithrombotic therapy, from DOACs to new antiplatelet agents, creates an urgent need for continuing professional development. Myths surrounding perioperative drug discontinuation are often deeply ingrained in clinical practice and need to be systematically replaced with current evidence. The near-universal demand for additional professional development is evident. In the same study, an overwhelming 96.4% of participants expressed a perceived need for more education regarding the management of this patient population [183]. Effective continuing professional development must address this gap by focusing on practical and actionable knowledge, how to interpret a patient’s medication list to assess pharmacological risk, how to apply advanced local hemostatic agents, and how to confidently communicate the rationale for continued therapy. This commitment to lifelong learning ensures that clinical decisions remain aligned with the latest evidence, thereby optimizing patient safety.

### 7.3. Value of Interdisciplinary Collaboration in Optimizing Patient Care

Although local hemostatic measures allow dentists to manage most patients independently, high-risk scenarios inherently require a team-based approach. Encouragingly, the data suggest that the instinct for collaboration is already present among trainees. In the survey conducted by Alabdulkarim et al. [183], the majority of participants (72.6%) indicated that they would consult a cardiologist before treating a patient using these medications. Furthermore, 76.9% stated that they would stop the medication only after consultation, and 70.8% would rely on local hemostatic techniques, a combined approach that aligns well with modern recommendations [183]. This interdisciplinary collaboration provides an important safety net, ensuring that the dentist’s surgical plan is fully integrated with the patient’s overall medical management.

## 8. Limitations of This Review

The limitations of this narrative review are primarily the variability of pharmacodynamics, which may influence recommendations for dental surgical treatment, as there is no clear definition of high bleeding risk. The small number of studies on patients with comorbidities requiring extensive surgical interventions is one of the major limitations, given the high number of patients under complex medication. Vulnerable populations, such as elderly patients, patients with renal failure, or combined medication, are often excluded from studies. The management of “bridges” in patients who have low-molecular-weight heparin as a medication remains controversial and insufficiently studied in dentistry. The need for patient assessment through interdisciplinary collaborations is one of the optimal approaches to the clinical case. The conclusions often remain descriptive and balanced, emphasizing the need for an individualized clinical decision-making process, which may be insufficient for a practitioner seeking clear guidelines.

## 9. Conclusions and Future Directions

Local hemostatic measures represent the main and definitive strategy for bleeding control. This intraoperative approach has reduced the number of severe complications that can occur both intraoperatively and postoperatively, allowing the dental field to align with the broader medical imperative of maintaining vital antithrombotic protection. Future research should focus on optimizing materials science on topical agents with prolonged release because managing the risk of bleeding, strictly related to systemic anticoagulation, remains one of the great challenges in dentistry. Reducing the number of hemorrhagic complications leads to good healing of the post-extraction wound, which ensures an optimal prosthetic field for subsequent interventions, such as implantation, for restoring the dental arches. As is well known, restoring the functions of the stomatognathic system is a priority in the patient’s life, contributing to improving the quality of life.

The academic and practical value of this review lies precisely in the clear identification of areas of uncertainty and discrepancies among the guidelines. This serves as a rational basis for the next crucial step: the implementation of local protocols for structured interdisciplinary collaboration. The future of this approach lies not in finding a single answer but in creating reliable decision-making frameworks that guide effective joint management between the prescriber and the surgeon, focusing on individualized risk assessment and local hemostasis measures as a fundamental pillar of any strategy.

## Figures and Tables

**Table 1:** Antithrombotic agents and dental extraction risk stratification.

Risk Level	Agent/Therapy	Key Impact	Management for Dental Extraction
Low	Single Antiplatelet (SAPT) Aspirin P2Y12 Inhibitor (e.g., Clopidogrel)	Minimal impact on hemostasis. No significant increase in clinically relevant bleeding.	Continue. Standard local measures suffice (pressure, sutures, gauze).
Moderate	Dual Antiplatelet (DAPT) Aspirin + Clopidogrel	Synergistic platelet inhibition. Increased bleeding risk, but manageable.	Continue (cardiac safety). Required: Planned robust local hemostasis (hemostatic socket sealants, TXA rinse).
	Direct Oral Anticoagulants (DOACs) Apixaban, Rivaroxaban (Xa inhibitors) Dabigatran (IIa inhibitor)	Direct, predictable anticoagulation. Higher immediate oozing vs. warfarin. Local agents do not reverse.	Continue. Schedule at trough. Use targeted local agents (e.g., TXA).
	Therapeutic LMWH	Significant anticoagulant effect.	Continue. Perform surgery at trough (≥12 h post-dose). Aggressive local measures.
High	Triple Therapy DAPT + Anticoagulant (e.g., Apixaban)	Maximal hemostatic impairment. Very high bleeding risk.	Mandatory specialist consult. Individualized plan; may briefly hold one agent. Maximal local hemostasis.
	High-Intensity VKA (INR > 4.0)	Uncontrolled anticoagulation.	Correct INR (low-dose vitamin K) > cessation. Postpone if elective. Aggressive local hemostasis
	Inherited Bleeding Disorder (e.g., Hemophilia on bypassing agents)	Unpredictable hemostasis. High delayed bleeding risk.	Pre-op Hematology consult. Coordinate factor replacement. Use antifibrinolytics (TXA). Meticulous technique.

**Table 2:** Pharmacokinetic profiles and perioperative timing recommendations.

Agent	Half-Life	Primary Elimination Route	Timing of Last Dose Before Procedure
Warfarin	36–42 h	Hepatic metabolism (CYP2C9, CYP1A2, CYP2C19, CYP3A4); minimal renal excretion	Continue (INR ≤ 3.5) or hold 3–5 days pre-op if INR is supratherapeutic
Apixaban	12 h	27% renal, 73% biliary/intestinal	Low risk: 24 h; high risk: 48 h
Rivaroxaban	5–9 h (young), 11–13 h (elderly)	66% renal, 28% feces	Low risk: 24 h; high risk: 48 h
Edoxaban	10–14 h	50% renal, 50% liver, and biliary/intestinal	Low risk: 24h; high risk: 48–72 h
Dabigatran	12–17 h	80% renal	CrCl > 50: 24 h (low risk), 72 h (high risk); CrCl < 50: 72 h (low risk), 120 h (high risk)
Aspirin	15–20 min (parent); platelet effect 7–10 days	Renal (urine)	Continue for low risk; hold 5–7 days before high-risk surgery
Clopidogrel	30–60 min (prodrug)	Renal (urine) and fecal	Continue for low risk; hold 5–7 days before high-risk surgery
Prasugrel	30–60 min (prodrug); 7 h (active metabolite)	Renal (urine)	Continue only after cardiology consult; hold 7 days before high-risk surgery
Ticagrelor	7–9 h; 6–12 h	Biliary elimination; urine/fecal	Continue for low risk; hold 3–5 days before high-risk surgery
Enoxaparin	4.5–7 h	Renal (40% of radioactivity, 8–20% of anti-Factor Xa activity recovered in urine at 24 h	12–24 h pre-op (at trough)

**Table 3:** Combined pharmacological and procedural risk assessment.

Procedural Risk ↓/Pharmacological Risk →	LOW (SAPT, Cilostazol)	MODERATE (DAPT, DOACs, Therapeutic LMWH)	HIGH (Triple Therapy, INR > 4.0, Bleeding Disorders)
Low (Single extraction, prophylaxis)	Standard local measures; continue therapy	Planned local hemostasis (Foundation Layer + TXA rinse).	Interdisciplinary consult; consider postponement if INR > 4.0
Moderate (Multiple extractions, single implant)	Enhanced local measures (Foundation + optional Augmentation)	Aggressive local protocol (Foundation + Augmentation + Stabilization).	Mandatory specialist consult; likely to be postponed for medical optimization
High (Full-arch surgery, complex grafting)	Augmented protocol with stabilization layer; consider timing optimization	Maximal hemostatic protocol; schedule at drug trough; interdisciplinary planning	Hospital-based setting; coordinate with cardiology/hematology; consider temporary modification only after risk-benefit analysis

## Data Availability

No new data were created.

## Abbreviations

The following abbreviations are used in this manuscript:

AGT	Anticoagulant therapy
APT	Antiplatelet therapy
INR	International Normalized Ratio
DOACs	Direct oral anticoagulants
NOAC	Non-Vitamin K Antagonist Oral Anticoagulants
LDA	Low-dose aspirin
DAPT	Dual antiplatelet therapy
VKAs	Vitamin K antagonists
FXa	Factor X
PAR	Platelet activation receptors
AT	Antithrombin
UFH	Unfractionated heparin
LMWH	Low-molecular-weight heparin
aPTT	Activated partial thromboplastin time
SAPT	Single-agent antiplatelet therapy
TXA	Tranexamic acid
ORC	Oxidized regenerated cellulose
